# Additively manufactured plastic plasma spectrometer

**DOI:** 10.1063/5.0219571

**Published:** 2024-11-20

**Authors:** Quetzal Larrick, Craig Pollock, Donald Hampton, Levon Avanov, Daniel Gershman, Denise Thorsen, Greg Shipman, Jesse Atencio, Anthony Melkomukov

**Affiliations:** 1 Space Systems Engineering Program, University of Alaska, Fairbanks, Alaska 99775, USA; 2 Denali Scientific, San Antonio, Texas 78248, USA; 3 Geophysical Institute, University of Alaska, Fairbanks, Alaska 99775, USA; 4 University of Maryland, College Park, Maryland 20742, USA; 5 Geospace Physics Laboratory, NASA/GSFC, Greenbelt, Maryland 20771, USA

## Abstract

We report results in the development and testing of a low resource tophat electrostatic analyzer (ESA) for space plasma measurements. This device has been additively manufactured (3D-printed) using fused deposition modeling. The classic tophat design is composed of four plastic pieces, without any surface coatings. The three conducting electrodes are printed from carbon nanotube infused polyether ether ketone (CNT-PEEK). The fourth piece, an insulating electrode support, uses pure PEEK. This ESA is designed to detect electrons in space from 10 eV up to 30 keV. We demonstrate that the printed CNT-PEEK is sufficiently electrically conductive to support the fast high voltage slewing often required for high time resolution measurements. The plastic ESA has been successfully vibrated beyond standard pre-flight levels, tested under keV electron beam illumination over a wide range of temperatures, and tested under UV illumination, simulating the solar Ly-α flux. In comparison with an identical machined aluminum ESA, our CNT-PEEK ESA provides nominal energy/angle bandpasses, closely matching simulation. These bandpasses imply minimal impact from surface charging at beam energies of 2–3 keV, although more investigation is needed. We also find that the CNT-PEEK ESA provides far superior out-of-band electron rejection and UV photon rejection compared to the machined aluminum ESA. We do not detect any problems with trapped gases or outgassing. This development offers the potential for significant mass savings, implementation of otherwise unattainable geometric configurations, and dramatic simplification in manufacturing and assembly processes required for the development of space plasma instruments.

## I. INTRODUCTION

Electrostatic Analyzers (ESAs) are common in Heliophysics missions and on planetary missions that feature an *in situ* space plasma component. *In situ* sampling of the local environment, including electromagnetic fields, plasmas, and energetic particles, is essential for many NASA heliophysics missions (National Research Council, 2013). An ESA is typically used in combination with electron multiplier particle detectors (Goodrich and Wiley, 1962) to measure local plasma fluxes in 3D energy/angle space. While an ESA selects for energy-per-charge and at least one dimension of angle, the particle speed is unknown without also measuring the particle’s mass-per-charge (m/q), which is sometimes included in a time-of-flight (Möbius *et al.*, 2016) or magnetic (Hoffman *et al.*, 1973) analyzer stage downstream of the ESA. However, in many space plasma applications, the charge state can be assumed to be ±1 (electron or singly ionized ion), and the mass can be assumed to be that of the electron for negatively charged particles or of the proton for positively charged particles [e.g., protons are known to comprise 96% of the ion particle density in the solar wind (Robbins *et al.*, 1970)]. If the particle m/q is measured or can be assumed, the ESA provides a measurement of the single particle distribution function [f(**v**)], which satisfies Boltzmann’s equation, and its velocity-space integral moments. These comprise the key fluid state variables of the plasma, enabling meaningful scientific studies of the plasma physics in process both at the site of the measurement and remotely from it (Fazakerley *et al.*, 1998).

In space flight, the size, weight, and power (SWaP) resources available to any instrument (like an ESA-based plasma instrument) are highly prized and limited. Therefore, strong motivation continually exists to minimize SWaP requirements. The recent proliferation of small spacecraft such as CubeSats has driven innovation in instrument design. We are motivated to make plasma instruments that are potentially viable on spacecraft as small as a 1-Unit CubeSat, which is a 10-cm cube of about 1–2 kg and consuming about 1–2 W (Johnstone, 2020). The prospect of flying constellations of such small spacecraft for targeted scientific purposes further motivates reductions both in instrument SWaP requirements and in developmental complexity.

ESAs are traditionally fabricated from aluminum using conventional machining, otherwise known as subtractive manufacturing (SM). We are exploring the use of Additive Manufacturing (AM), popularly referred to as 3D printing, for space flight instrument fabrication, beginning with the design and AM-fabrication of ESAs. The use of AM rather than SM holds the potential to simplify instrument development and reduce instrument SWaP requirements via design elements not available using SM techniques. AM may enable the manufacture of a “monolithic” ESA—one solid part with no internal fasteners required. This approach will reduce variability in the manufacturing and assembly processes and greatly simplify the development process by eliminating interfaces between parts of the ESA. Finally, use of AM with appropriately selected materials may offer both enhanced electro-optical performance and functional design elements not practical to fabricate in the SM environment. AM can be performed with metals, plastics, or even ceramics, but this work is concerned only with plastics.

**FIG. 1.**
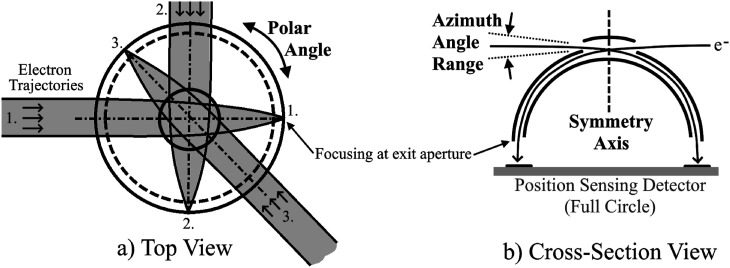
Tophat ESA conceptual diagrams: Top view (left) and cross section (right). Azimuth and polar angles referred to in the text are indicated. (Reproduced from C. W. Carlson *et al.* “An instrument for rapidly measuring plasma distribution functions with high resolution,” Adv. Space Res. **2**, 67–70 (1983) with the permission of Elsevier.).

Here, we focus on the “tophat” ESA (Carlson *et al.*, 1982; hereafter denoted C83), a popular ESA configuration (e.g., Johnstone *et al.*, 1997; Johnstone *et al.*, 1987; Reme *et al.*, 1997; Young *et al.*, 2004; McFadden *et al.*, 2009; Pollock *et al.*, 2016; and McComas *et al.*, 2017) that we illustrate in Fig. 1. The “tophat” nomenclature is somewhat colloquial in the broader scientific community, and we differentiate between a tophat, which is a type of entire ESA, and a “top cap,” which is one of a tophat’s three electrodes, as is described subsequently. We have designed a tophat ESA whose size and shape can be accommodated by a standardized protrusion from a CubeSat within the “tuna can,” a cylindrical volume of 6.4 cm diameter × 3.6 cm length. This volume is normally unused and lies within the interior of the coil spring used to eject a CubeSat from its host deployer. Alternatively, this ESA’s cylindrical shape is also designed to fit within an ejectable sub-payload for a sounding rocket.

The main unusual feature of this ESA is that it is fully fabricated from polyether ether ketone (PEEK). PEEK is a high-grade engineering plastic often used in electrically insulating components in space flight applications, particularly in high voltage applications and those where molecular contamination of sensitive optical and detection surfaces must be avoided. The exclusive use of plastics for ESA fabrication is new, although plastics with coatings have been used in space before (Fedorov *et al.*, 2011). Normally, metals such as aluminum are required for the conducting electrodes and grounded chassis elements in an ESA to impose electric fields and drain deposited charge away. New Carbon Nanotube (CNT)-infused high temperature engineering plastics like PEEK (Mohiuddin and Van Hoa, 2011) have useful electrical conductivity, enabling exploration of their use as electrodes in plasma instruments. The additional development of AM printers able to print high-temperature engineering plastics (Das *et al.*, 2020) enables the fabrication of complete ESAs from a combination of insulating and conductive PEEK. The conductivity of CNT-PEEK is low compared to metals but still sufficient for draining away charge. While a metal might have a resistivity of ∼10^−6^ Ω cm, CNT-PEEK can be in the range of ∼10^1^ Ω cm (Goncalves *et al.* 2018) and can have a discharging time constant on the order of a microsecond based on our analysis.

The rest of this paper is organized as follows: In Sec. II, we describe more detail about ESAs and specifically the simulated parameters of our CNT-PEEK ESA. In Sec. III, we describe the AM fabrication process and some lessons that we have learned along the way. In Sec. IV, we describe performance testing under both electron beam and UV photon illumination in high vacuum and summarize environmental testing that has been performed. In Sec. V, we present our conclusions, address some pitfalls that may present themselves, and briefly discuss future developmental directions.

## II. DESIGN OF THE ESA

**FIG. 2.**
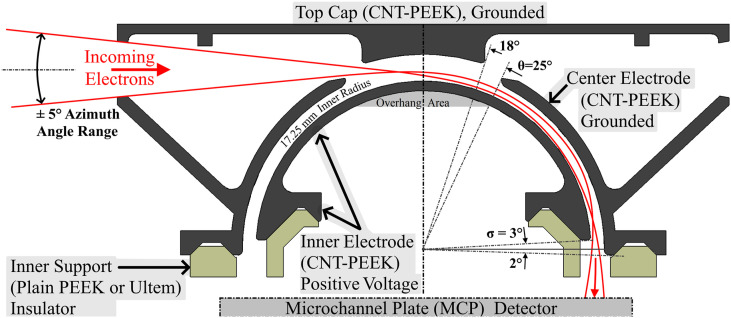
CNT-PEEK ESA section view from Solidworks^®^ CAD showing the four main pieces and conceptual electron trajectories.

An ESA is composed of a pair of concentric curved conducting electrodes that are electrically biased with respect to one another so that a near-uniform electric field points from one plate to the other across the narrow, curved gap between them (see Fig. 2). ESAs are deployed with one end of the curved gap (the entrance aperture) viewing the plasma and the other end (the exit aperture) facing a particle detector. A particle that enters the gap between the electrodes is deflected by the applied electric field. If the particles’ kinetic energy/charge (E/q) is such that the electric field force is nearly equal to the centripetal force required to keep the particle on a circular trajectory between the plates, then it will impact the particle detector and be counted. If the particle’s E/q is significantly higher or lower, it will impact the electrodes and be absorbed before it can reach the detector.

**FIG. 3.**
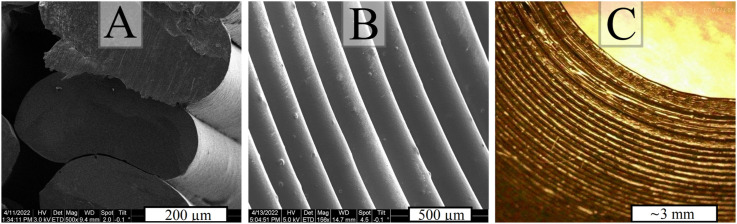
Views of the printed ESA. A and B are from a scanning electron microscope. A shows a cut through several layers, showing where the extruded lines of plastic bond together. B shows the most overhanging part of the center electrode, where each bright line is the seam between plastic extrusions. C shows the same surface as B at a larger scale, through an optical microscope.

**FIG. 4.**
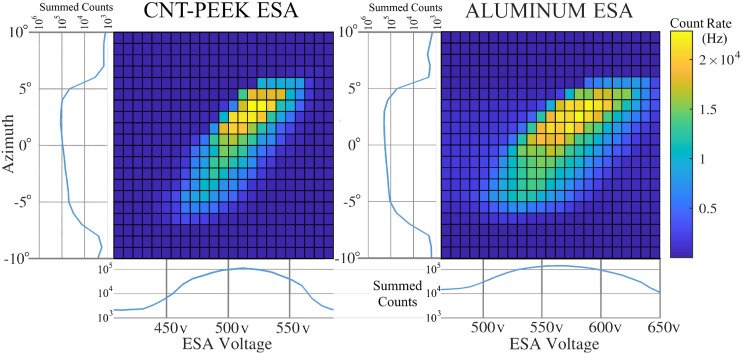
Energy-angle scans for CNT-PEEK and aluminum ESAs, with one-D angle and voltage distributions obtained by summing over the neglected dimension in each case. Responses of ESAs made from CNT-PEEK (left) and aluminum (right) are compared. The voltages are not the same.

The “tophat” configuration shown in Fig. 1 or some variation of it has become nearly ubiquitous in scientific *in situ* space plasma measurements for particle energies of <1 eV up to tens of keV. The C83 tophat ESA features two concentric and nearly hemispheric electrodes: the inner electrode and the center electrode. A grounded outer electrode, often referred to as the top cap, is added above the entrance aperture in the center electrode to contain the applied electric field and guide arriving particles into the curved gap. Charged particles within a narrow range of energy-per-charge (E/q) and azimuth angle freely fly through the gap between the inner and center electrodes and emerge at an annular exit aperture near the hemispheric ESA’s equatorial plane. This is illustrated in the simulation results shown in C83’s Fig. 3, as well as this paper’s Fig. 4. The center of the E/q bandpass is directly proportional to the voltage applied to the inner electrode. The constant of proportionality, often referred to as the analyzer ratio or k-factor, depends nearly exclusively on the ratio of the gap (Δ_1_) between the inner and center electrodes to the radius (R_1_) of the inner electrode. The inner electrode is biased with positive voltage to attract negatively charged particles (e.g., electrons) or negative for positively charged particles (e.g., protons). The center electrode and top cap are both typically grounded. The tophat ESA admits particles within a cylindrically radial field-of-view near a plane parallel to its equatorial plane and directs those that are transmitted to an annular detector area, typically the outer portion of a circular microchannel plate (MCP) detector. Particle detection implies the particle’s E/q, given knowledge of the voltage applied to the inner electrode at the time of detection. One-dimensional position sensing around the annular detector’s circumference determines the particle’s arriving polar angle (see Fig. 1). Therefore, with a single applied voltage and a fixed ESA orientation in space, the flux of particles arriving near a single E/q and azimuth angle (see Fig. 1) can be measured as a function of polar angle. Controlled sweeping of the applied ESA voltage provides sequential sampling of multiple E/q values, admitting measurement of a 2D (E/q, polar angle) particle count spectrum near a fixed azimuth angle. Measurement of the full 3D velocity space is most commonly achieved by mounting a tophat ESA on a spinning spacecraft, as in the Hot Plasma Composition Spectrometer (Young *et al.*, 2016) on NASA’s Magnetospheric Multiscale (MMS; Burch *et al.*, 2016). Alternatively, pitch angle measurements can be obtained without spinning if the spacecraft remains oriented so that the magnetic field vector is perpendicular to the ESA’s symmetry axis. More rapid 3D velocity space measurements were enabled in the case of the MMS Fast Plasma Investigation (FPI; Pollock *et al.*, 2016) by placing a large number (eight for electrons and eight for ions) of tophat ESAs each viewing different planes in velocity space. More rapid or comprehensive measurements can also be made by mechanically or electrostatically scanning the field of view, or with a design of ESA that captures a wider field of view instantaneously, such as FIPS on the MESSENGER spacecraft (Andrews *et al.*, 2007), or the designs in (Vaisberg *et al.*, 2001 and Morel *et al.*, 2017).

The ESA was designed based on the study of C83 and is optimized for measuring auroral electrons in the E/q range between 10 eV and 30 keV. Figure 2 shows a cross section of the design, which was modeled in SolidWorks and optimized based on SIMION™ electron ray tracing. Critical design parameter selections as defined by C83 are listed in Table I.

**TABLE I.** ESA design parameters and predicted performance based on SIMION and C83.

Name	Comment	Value	Unit
R_1_	Radius of inner electrode	1.72	cm
R_2_	Radius of center electrode	1.86	cm
R_3_	Radius of outer (top cap) electrode	2.00	cm
Δ_1_	Gap between inner and center electrodes	0.138	cm
Δ_1_/R_1_	Controlling performance ratio	0.08	⋯
θ	Optimal ½-angle subtended by hole at pole of center electrode	17.5	deg
	(As built)	25	
σ	Optimal truncation angle of hemispheres near the equatorial plane (from C83)	9	deg
	(As built)	3	
G	Predicted geometric factor (from C83)	0.0060	cm^2^ sr eV/eV
	Predicted geometric factor (from SIMION ray tracing)	0.0073	
ΔE/E	Electron energy bandwidth FWHM (from SIMION)	21%	⋯
ΔAz	Polar angle bandwidth FWHM (from SIMION)	9.4	deg
A.R.	Analyzer ratio (k-factor): Ratio of electron energy to ESA voltage. (Theoretical)	6.25	⋯

We attempted to design an ESA with relatively generic design parameters to be relevant for most science applications. The ratio Δ_1_/R_1_ controls the tophat ESA performance and, along with the radius of the ESA, its sensitivity as described in C83. For a fixed Δ_1_/R_1_, the instrument sensitivity scales with R_1_^2^ (a larger instrument yields more collection area at the entrance aperture). For fixed R_1_, the entrance aperture and both the energy and azimuth angle bandpasses expand with increasing Δ_1_, yielding greater sensitivity. This comes at the expense of both reduced analyzer ratios, limiting the upper energy/charge range for a given high voltage capability. In addition, a larger Δ_1_ causes greater vulnerability to noise from solar Ly-a UV radiation and the photoelectrons that they produce, penetrating to the detector by reflecting from ESA electrodes, which are more widely spaced apart.

The ESA design calls for only one insulating part, the “inner support” that mounts other ESA parts and provides high voltage (up to 5 kV) isolation between the inner and center electrodes. Instead of PEEK, this part was temporarily printed in another high-performance plastic called PEI (Ultem) due to limited material availability for some of the testing.

## III. ESA FABRICATION

CNT-PEEK ESAs designed as described earlier have been fabricated at the University of Alaska Geophysical Institute using an Intamsys Funmat HT printer. This printer features exceptionally high temperatures on the nozzle, printing bed, and build chamber, necessary for high performance plastics like PEEK and PEI. Parts were printed with a nozzle temperature of around 420 °C, although the true temperature of the plastic is somewhat colder while it is being extruded. The build chamber was operated at 90 °C and the printing bed at 140–160 °C. The high chamber and bed temperatures improve plastic adhesion and reduce warping or cracking of the print. Nano polymer adhesive from the company Vision Miner was used to improve adhesion to the carbon fiber printing bed. Remaining adhesive on the parts was sanded away before vacuum testing to ensure it would not alter the final material properties.

The layer-by-layer AM process presents difficulty in overhang areas where material must be extruded above empty space, especially when the overhang angle exceeds 60° from vertical. This ESA design contains severe overhangs on the inner and center electrodes, highlighted in Fig. 2 as “Overhang Area.” Temporary supporting material was printed below such overhangs, to be removed in post-processing. Only a single-nozzle printer was available for this work; therefore, the support material had to be the same plastic as the main part. When properly printed, support material only lightly adheres to the main part, so we manually removed it with hand tools. To achieve consistent surface quality, we avoided using support material under the electrode surfaces, which guide electrons through the ESA. This posed a challenge for the center electrode, where there are overhangs at up to 65° from vertical near the entrance. For these unsupported but optically important overhanging areas, we tried numerous slicing software settings and found success with the use of low nozzle movement speed, aggressive cooling, and unusually wide extrusions (∼0.4 mm) at 0.15 mm layer thickness. The print time required for all parts of our ESA was under 8 hours. In the future, we intend to use a multi-headed printer and soluble support material. Soluble support material will allow overhangs to be reliably printed with better surface quality. Figure 3 shows three microscope views of printed ESA parts. The naturally occurring ridges of the printed surface shown in Fig. 3 are encountered by electrons and UV photons as they enter the ESA. We believe this surface has a serendipitous effect of acting like optical roughening or serrations to reduce UV and out-of-band electron transmission.

The ESAs tested in this document were not built to spaceflight quality standards, and high-precision positioning or alignment of the parts during assembly was not a priority. Post-processing time for an ESA was only a few hours, including support material removal, hole drilling/tapping, cleaning, and hand-fitting the screw interfaces. Though it is feasible to post-process a printed part with machine tools if extremely accurate surfaces or holes are required, we did not do so. The printer was not operating at its limits of accuracy or surface finish for these ESA parts. To achieve better manufacturing accuracy and lower surface roughness at the expense of time, thinner layers with lower print speeds and more narrow extrusions can be used. The ultimate limits of additive manufacturing are still unknown. While the basic process of plastic extrusion may never be as repeatable as metal milling, we predict that the overall electrode alignment might be more accurate with a “monolithic” printing technique. Compared to traditional methods, a monolithic ESA would avoid all assembly errors and error-prone mechanical interfaces because it would be one fused-together piece of plastic, combining all the electrodes and insulators.

## IV. PERFORMANCE AND ENVIRONMENTAL TESTING

Testing was performed both at the University of Alaska Fairbanks (UAF) and at NASA’s Goddard Space Flight Center (GSFC). The GSFC facility has a well-established history of calibrating such space flight instruments, e.g., the dual electron spectrometers of the FPI instruments (Pollock *et al.*, 2016) now flying on NASA’s MMS (Burch *et al.*, 2016) mission. The test program is summarized in Table II, where each test and its results are described below.

**TABLE II.** Summary of testing and results.

Test	Location	Results	Conclusions
CNT-PEEK resistivity estimate	UAF	10^4^–10^6^ Ω measured between screws in the plastic, depending on several uncontrolled variables and print settings	Nominally sufficient. Updated plastic formula and printing techniques may be needed to achieve lower resistivity
ESA voltage sweeping	UAF	The printed ESA detects an electron beam at the same voltages during 100 Hz voltage sweeps as during steady-state operation	The ESA is conductive enough to behave electrostatically like a metal ESA at high voltage sweeping rates
Energy-angle scans	GSFC	Plastic ESA yields a bandpass similar to metal with reduced out-of-band electron transmission	Printed CNT-PEEK ESA electron optics work more ideally than metal ESA
UV transmission	GSFC	Printed CNT-PEEK ESA detects 100× less UV than aluminum	Printed CNT-PEEK surface is roughly 10x less reflective than aluminum at 121 nm. Surface blackening coatings may not be needed for certain designs
UV photoelectrons	GSFC	The printed CNT-PEEK shows higher-energy photoelectrons, implying its work function is significantly smaller than that of aluminum	This is unfortunate, but despite the lower work function, CNT-PEEK photoelectron counts are still much less than aluminum
High-flux electron beam	UAF	ESA behaves almost identically when exposed at high and low beam fluxes at 2 keV	The CNT-PEEK ESA can absorb significant plasma current onto its surfaces without influencing performance at 2 keV
ESA temperature testing	UAF	ESA voltage bandpass remains consistent across temperatures, even at high beam flux	The CNT-PEEK ESA will work well across the range of normal earth-orbiting thermal environments
RGA/outgassing	GSFC	No signs of excessive outgassing or contamination	This printed PEEK probably does not pose a molecular contamination threat to MCPs
Vibration	UAF	No damage after 20-G GEVS profile for 20 minutes in each of two orientations	A printed PEEK ESA will survive rocket launches

### A. Resistivity analysis

The first tests of printed CNT-PEEK were conductivity measurements using ordinary handheld multimeters and a Samyo VC60B+ high-voltage insulation testing multimeter. Resistances across the surface are difficult to measure accurately because the probe’s contact geometry with the surface has a dramatic influence on the readings. To avoid this uncertainty, we measured resistance between #0–80 (∼1.5 mm) sized steel screws inserted in threaded holes on the outer rim of the ESA’s center electrode. Measured resistance values extended from less than 10 kΩ to 1 MΩ, depending on factors including part geometry, printing conditions, and screw tightness. We crudely estimate the volume resistivity is on the order of 10^4^ Ω*cm or less. Charging time constant calculations and a practical demonstration to support the plastic’s viability are shown in Sec. IV C titled “ESA Voltage Sweeping.”

In an extreme environmental charging case of only electrons impacting the ESA’s top cap with a number density of 40 cm^−3^ and speed of 1000 km s^−1^ (flux = 4 × 10^9^ electrons cm^−2^ s^−1^) and a top cap resistance of 1 MΩ, the top cap would still only be expected to charge at 0.03 V relative to spacecraft ground. Such a voltage error would likely only harm the ESA’s performance at energies below about 1 eV. While this appears to be a worst-case analysis, we are assuming a homogeneous conductivity, which may be unrealistic for this case of carbon nanotubes embedded in an insulating material.

We investigated conductivity variation over temperature by placing a printed CNT-PEEK inner electrode in a laboratory freezer. Resistance measured screws on the center electrode was 40 kΩ at +20 °C and 100 kΩ at −80 °C. Clearly, temperature does influence the conductivity significantly, in a similar pattern as a semiconductor, but the increased resistance is likely still acceptable. While the bulk conductivity is sufficient, surface conductivity on a microscopic scale is not fully resolved. A critical question remains as to whether microscopic insulating islands might appear on the material surface, especially at cold temperatures, causing localized surface charging sufficient to interfere with the transmission of low energy (<100 eV), as has been hypothesized in (Fedorov *et al.*, 2011). This is further discussed in Chapter 5.

### B. Electron energy-angle scans

A spatially broad (∼8 cm wide) electron beam was directed toward the ESA. The beam energy was 3 keV, and Helmholtz coils were in use, canceling about 90% of the Earth’s magnetic field, so any magnetic curvature of the beam was negligible. Transmitted electrons impacted an MCP detector whose output pulses were counted so that each voltage-azimuth angle combination has an associated count rate measurement. This test was performed in the GSFC chamber on an automatic motion control stage that swept the azimuth angle from −10 to +10° in 1° steps. At each angle step, the ESA voltage was stepped in ∼10 V increments over the full ESA voltage bandpass. Variations of beam flux throughout the experiment were measured with a Faraday Cup but found to be negligible. The results shown in Fig. 4 correspond to a single polar angle, equidistant between two of the ESA’s top cap supports. The data show that the overall energy-angle bandpass shape is very similar for the CNT-PEEK and metal ESAs, as expected. The bandpasses are centered at slightly different voltages, probably owing to manufacturing and assembly differences between the metal and CNT-PEEK ESAs. Evidently, the metal ESA has a somewhat wider gap between the electrodes, causing higher voltages to be required to transmit the 3 keV electrons. The CNT-PEEK ESA’s energy bandpass is more narrowly confined compared to that of the Aluminum ESA. This more severe out-of-band electron transmission is almost certainly caused by electrons that have collided with one of the ESA electrodes but were still scattered forward, eventually reaching the detector when they should have been absorbed. We expect that the CNT-PEEK has a reduced secondary electron yield compared to metal because it has a rougher surface and because it is primarily composed of lighter atoms (carbon and hydrogen), as opposed to a metal like aluminum. Accurately inspecting the dimensions and surface properties of the ESAs was not within the scope of this work and, therefore, it is difficult to quantitatively compare properties based on Fig. 4’s results. After normalizing for the different beam currents, the metal ESA had 1.46x higher total counts across the entire energy-angle scan, but the analyzer ratio was 9% smaller. These are likely related, indicating that the metal ESA probably had a larger gap between the electrodes. According to (Carlson *et al.* 1982)’s Fig. 6, we expect a 9% smaller analyzer ratio to correspond with a 25%–30% increase in sensitivity (geometric factor). However, we are not able to conclude whether all the differences are due to manufacturing and assembly, or due to surface roughness, or potentially due to a fundamental difference between the materials. The roughly 0.1 mm-sized ridges that form due to the printing layers may alter the PEEK ESA’s electron optics.

**FIG. 5.**
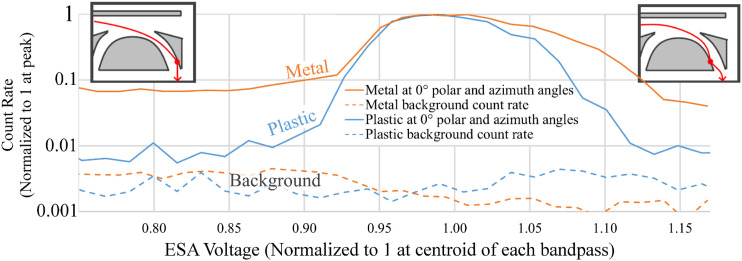
Out-of-band electron comparison from the GSFC energy-angle scans. The vertical axis is log-scaled to show the “tail” off both sides from the peak ESA count rates.

Figure 5 shows a comparison between the CNT-PEEK and Aluminum ESA E/q bandpasses after summing across azimuth angle and placing them on common and independently normalized energy and count rate scales. This graph is created from the same GSFC data as is shown on the bottom of Fig. 4. The CNT-PEEK and aluminum bandpasses are similarly shaped, falling off more steeply at low voltages than at higher voltages. There are, however, significant differences between the two materials. The aluminum ESA response has a broader peak and exhibits an out-of-band response at nearly 10% of the peak count rate, even when at only 75% of the peak voltage. The CNT-PEEK ESA has a narrower bandpass and exhibits an out-of-band response of under 1% of the peak count rate, which is near the detector background noise level. The signal-to-noise ratio for these measurements was limited to only about 200 because of MCP dark counts. The increased out-of-band response of the aluminum ESA is undesirable because it represents a reduction in energy resolution and a source of electron energy ambiguity. An ideal ESA would absorb those out-of-band electrons such that none reach the detector. This result implies that the CNT-PEEK surfaces have the desirable effect of reduced forward scattering of electrons and perhaps also reduced forward emission of secondary electrons. This is probably caused by the surface roughness from 3D printing layers but might also be caused by an intrinsically low secondary electron yield. However, the aluminum ESA was not coated in this comparison.

### C. ESA voltage sweeping

For many space-flight plasma applications that demand precise time and position resolution of electron energy spectrum measurements, the ESA must rapidly sweep through a wide range of voltages. Voltage sweeping is necessary to provide electron spectra covering several energy decades many times per second. We tested the CNT-PEEK ESA with a three-decade exponentially decaying voltage sweep at 8 ms cadence (sweep time constant ≈1 ms). The voltage retrace time at the end of each sweep was ∼1 ms, and the sweep repetition rate was 100 Hz. For the somewhat resistive ESA electrodes to effectively follow the power supply’s driving voltage, their R * C time constant must be short compared to the power supply voltage variations. This should be achievable with the CNT-PEEK ESA given its <1 MΩ resistance and ∼10 pF capacitance that yield a time constant on the order of 10 *µ*s. Therefore, the ∼1 ms voltage retrace time is long enough for 100 ESA R * C time constants to occur, ensuring that the ESA voltage is not significantly “lagging behind” its power supply.

**FIG. 6.**
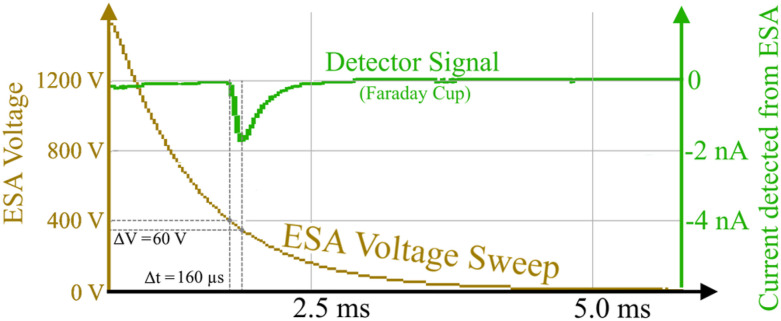
Fast CNT-PEEK ESA voltage sweep (tan, scale on left) and Faraday cup detector response (green) in the presence of a 2 keV electron beam.

Tests of ESA electron transmission at UAF used a Faraday cup directly under the ESA instead of an MCP detector because we did not have a MCP detector available at UAF. The negative Faraday cup signal seen in Fig. 6 represents the transmission of electrons through the ESA. The Faraday cup we used has no electron multiplication, so it required a much more intense electron input to produce a measurable output than an MCP detector. Therefore, the UAF tests used fluxes of 10^10^–10^12^ electrons cm^−2^ s^−1^, while the GSFC tests used only 10^5^–10^6^ electrons cm^−2^ s^−1^. Tests at UAF also used a 2 keV electron beam, while those at GSFC used 3 keV. Figure 6 shows the Faraday cup current signal responding as the ESA voltage sweeps through a certain ESA voltage bandpass. The Faraday cup was placed directly below the ESA’s exit aperture, so it was measuring only the electrons that the ESA allowed through.

**FIG. 7.**
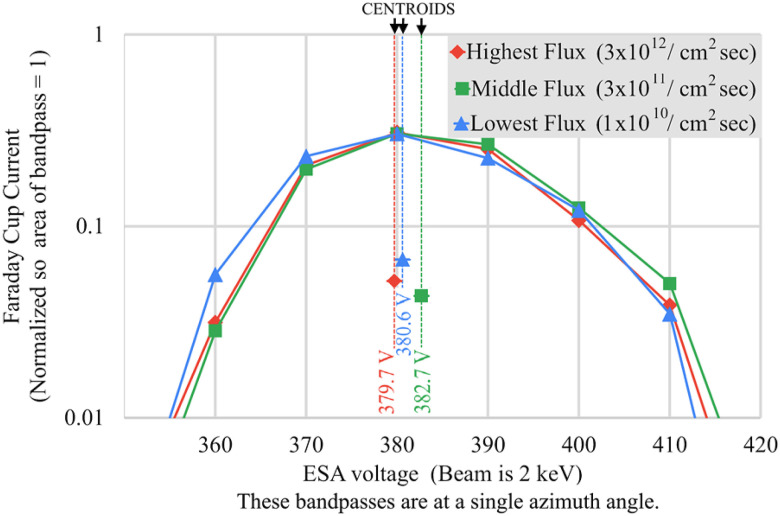
Printed ESA voltage bandpasses at various beam fluxes.

The electron beam energy was maintained at 2 keV, and the Faraday cup collected measurable current only when the ESA voltage swept through the ESA’s E/q bandpass. The Faraday cup is seen to have collected a significant signal when the ESA was between 410 and 350 V, matching closely with the range of constant (non-sweeping) voltages where this ESA detects a 2 keV beam, as seen in Fig. 7 below. This similarity in performance between steady-state and fast sweeping demonstrates that the CNT-PEEK ESA can be used to acquire electron energy spectra at high voltage sweeping rates and, therefore, at fine time resolution on the order of 100 Hz or more. The exponential decay seen after the detector signal peak in Fig. 6 is dominated by the Faraday cup circuitry, not the ESA. The Faraday cup preamplifier intentionally had a high R * C, so it was acting as an integrator.

### D. High-flux beam testing

Accumulation of charge from the space environment onto insufficiently conducting surfaces of an ESA-based plasma instrument can disrupt the measurements. High-flux testing was performed at the UAF Space Systems Engineering Laboratory in a thermal vacuum chamber to test for surface charging effects under high beam flux conditions and extreme temperatures. Results of high-flux testing at room temperature are shown in Fig. 7. Even the lowest beam flux among the three fluxes tested is several times more intense than that of an extreme hypothetical situation of a solar wind consisting of only electrons with a number density of 40 cm^−3^ and bulk speed of 1000 km s^−1^, carrying an electron flux of 4 × 10^9^ cm^−2^ s^−1^. A complete analysis of the worst-case charging conditions has not been performed. Some locations such as the magnetosheath may have more intense fluxes. This requires additional analysis of electrons and ions across all energies, plus photoelectrons caused by solar radiation of all energies.

Only a 3 V (∼1%) shift in the bandpass centroid was seen when comparing the high flux and low flux measurements. Consistent performance at various intense beam fluxes indicates that the CNT-PEEK is sufficiently conductive to dissipate charge collected from the local environment at room temperature, at least for use with higher energy electrons. All UAF beam tests were performed with 2 keV electrons in a Helmholtz cage. However, we still encountered excessive magnetic deflection below 500 eV and were unable to collect consistent measurements because the chamber and stepper motors were still dramatically altering the magnetic environment. To fully address surface charging concerns at lower electron energies, tests with better magnetic cancellation are necessary. Alternatively, tests with a beam of much more massive ions instead of electrons will allow dramatically reduced magnetic disturbance errors, simply requiring the ESA and electron gun voltage polarities to be reversed.

### E. Beam testing over temperature

**FIG. 8.**
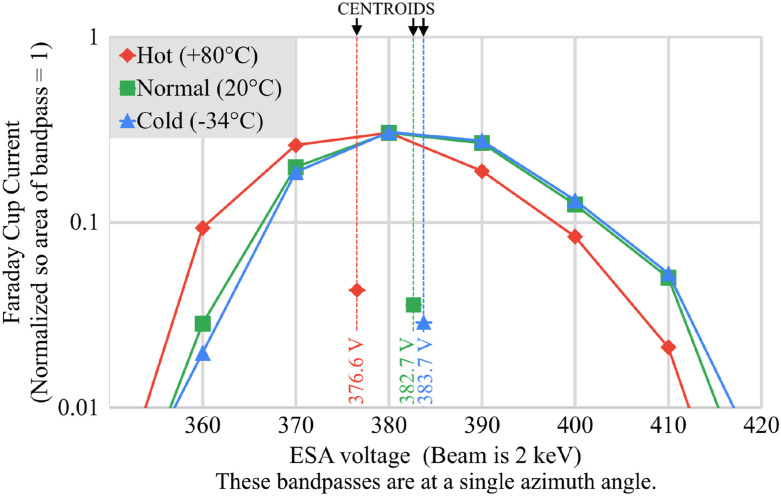
Printed ESA voltage bandpass at various temperatures.

The CNT-PEEK ESA performance was tested over temperature in a thermal vacuum chamber. The chamber used a heat exchanger plate to heat or cool the ESA and its mounting fixture. To improve the chamber’s ability to cool the ESA, it was surrounded by a sheet aluminum shroud to reduce radiative heating from the chamber walls. The temperature of the ESA was measured using a thermocouple attached near the mounting screws of the ESA. Figure 8 shows the results of three tests at hot, normal, and cold temperatures, which were all conducted at beam fluxes within ±50% of 3 × 10^11^ cm^−2^ s^−1^. A small shift (∼6 V or 1.5%) of the ESA voltage bandpass was seen at +80 °C (red line). While it is possible that an electrical change in performance occurred, this bandpass shift may have also been caused by thermal expansion or warping of the ESA and/or its mounting fixture. Plain PEEK’s coefficient of thermal expansion of ∼45 ppm/K is roughly twice that of aluminum, although we do not have data for this specific CNT-PEEK.

### F. UV photons

Ultraviolet (UV) photon testing was performed to determine how effectively the ESA suppresses photon transmission to the detector. This is important because far-ultraviolet photons produced by the Sun (primarily Lyman-α at 121 nm) can trigger an MCP detector and represent an important or even debilitating noise source. UV photon testing was performed in the same GSFC testing chamber as were the energy-angle scans shown in Figs. 4 and 5. The Krypton gas UV source produces two emission lines very close to 121 nm. Based on prior calibrations with a photodiode, the UV flux was estimated to be 6 ± 2 × 10^11^ photons cm^−2^ s^−1^, slightly greater than the solar Lyman-α flux near Earth. This photon testing was intended only to measure the direct transmission of scattered photons but not photoelectrons. To ensure photoelectrons were rejected, we operated the ESA at +400 V, sufficient to attract any photoelectrons on to the inner electrode and prevent them from reaching the detector. Any photoelectrons must have relatively low energies of less than 11 eV, the value of a 121 nm UV photon’s energy.

**FIG. 9.**
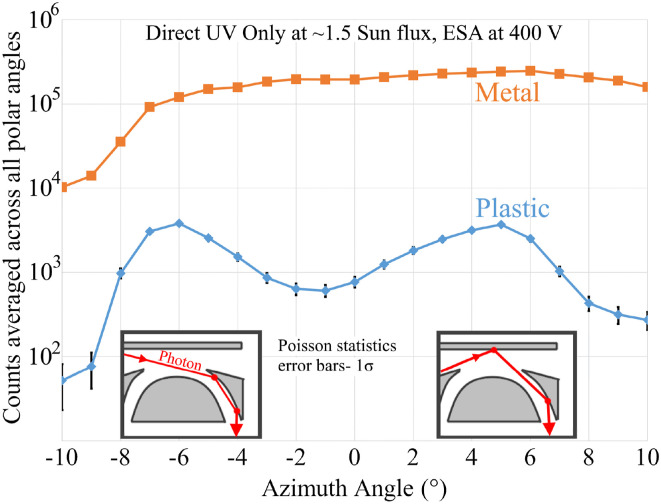
Comparison of count rates vs azimuth angle for ESAs made from CNT-PEEK (blue) and aluminum (orange) under laboratory-simulated solar Ly-α illumination. The insets illustrate hypothesized two-bounce paths producing the two peaks in the CNT-PEEK ESA response.

Figure 9 shows the UV-induced count rate plotted vs incident azimuth angle (see Fig. 1) for the CNT-PEEK ESA (blue line) and the aluminum ESA (orange line). The aluminum ESA shows a relatively large and uniform UV contamination across polar angles. In contrast, the CNT-PEEK ESA shows substantially reduced response and is non-uniform, displaying two distinct peaks near −6° and +5° polar angles.

Based on the results of a simple MATLAB ray tracing script, we believe the main source of UV counts and the two-peak structure in the CNT-PEEK ESA response is due to two families of two-bounce paths available for photons to reach the detector through the ESA, sketched as insets in Fig. 9. One family of paths near +5° includes reflections first from the top cap and then from the center electrode. The second family, near −6°, includes two reflections, both from the center electrode. It is possible to eliminate these two-bounce paths by extending the inner and center electrodes slightly beyond the ESA equatorial plane. A trade is required, however, because this truncation angle (σ in Fig. 2) controls the quality of focus for incoming parallel trajectories at the detector. We wish to extend the electrodes sufficiently to eliminate the two-bounce photon paths while not unduly degrading the ESA’s polar angle resolution. A potentially similar situation of UV suppression and carbon-based material was on the MIMA instrument (McCann *et al.*, 2007). They used machined graphite for portions of the instrument to suppress UV and secondary electrons. They also used a “photon trap” near the ESA entrance made of thin metal plates parallel to the incoming photon paths. We plan to combine those ideas and 3D print complex light-trapping structures with a naturally UV-absorptive plastic.

When integrated over the 2D angular field of view, our UV measurements show that this CNT-PEEK ESA rejects UV photons ∼100x more effectively than the uncoated aluminum ESA we tested. However, aluminum ESAs for space flight are typically fabricated with serrations machined into their electrode surfaces and have those surfaces coated with materials such as Copper Sulfide Black (Cu_2_S) or gold black to reduce UV transmission. The superior inherent rejection of the CNT-PEEK ESA may, with optimizations like those described earlier, be sufficient to allow its effective use without resorting to the application of black absorptive coatings on the ESA electrodes, a costly and delicate process in the flight instrument development cycle.

The UV photon rejection is likely due to a combination of material properties and the surface texture. The 3D printed surface naturally has ridges between printing layers, which likely trap photons that reflect at shallow angles. Similarly shaped serrations are intentionally machined into many metal ESAs for UV rejection. Although we did not directly measure the CNT-PEEK’s single-bounce UV absorption, it can reasonably be expected to be much higher than metals. Hydrocarbon polymers are generally known for absorbing UV, and PEEK is no exception. Absorptivity in the range of 90% was seen with a PEEK film by Jolly, 2019. Carbon nanotubes are also effective at absorbing many wavelengths of light. The famous coating “VANTABLACK” achieves exceptional absorptivity from carbon nanotubes. We expect that further increasing the concentration of nanotubes will yield an even higher UV absorptivity beyond that of plain PEEK.

### G. Photoelectrons

**FIG. 10.**
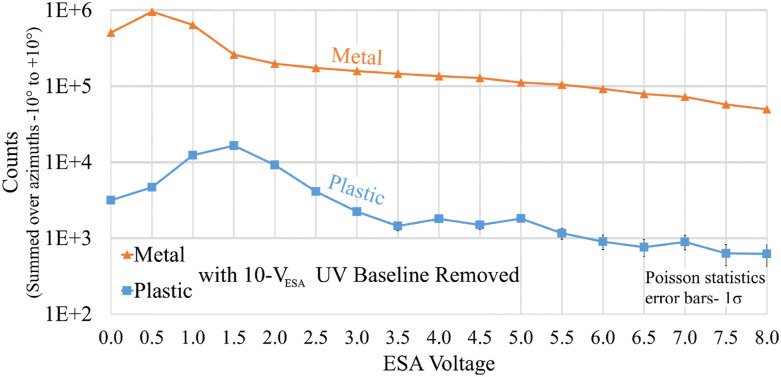
UV photoelectrons comparing CNT-PEEK and aluminum ESAs. Data are integrated across azimuth angles over the range (−10°, 10°).

This test is similar to the UV direct transmission test described earlier, except that the ESA is operated over the range of 0 to +5 V instead of fixed at +400 V in order to perform electrostatic analysis of any emitted photoelectrons. The background level of direct UV was assumed to be at an ESA voltage of 10 V because 10 V should be enough to attract and absorb the vast majority of 121 nm photoelectrons before they reach the detector. Figure 10 shows the count rates with the 10 V_ESA_ baseline level subtracted. Figure 10 demonstrates that the CNT-PEEK and aluminum ESAs both exhibit count rate peaks at a certain low ESA voltage, indicating the presence of significant photoelectrons. However, the peaks are found at different voltages. The CNT-PEEK photoelectrons peak near 1.5 V_ESA_, while those from the aluminum ESA peak near 0.5 V_ESA_. This may imply that the photoelectrons emitted from the CNT-PEEK were considerably more energetic than those emitted from the aluminum and, by extension, that the CNT-PEEK has a lower work function than that of the aluminum. However, this implication is questionable because we do not know where in the ESA these photoelectrons were emitted and, therefore, the extent of electrostatic filtering to which they were subjected. While a lower work function may turn out to be a disadvantage of CNT-PEEK, the total photoelectron count rate was still 53× smaller in the CNT-PEEK ESA than the aluminum ESA, and noise from photoelectrons represents a small contribution, localized near a few eV, compared to the potentially much larger signal from direct photons that extends uniformly across the entire ESA energy range. For both materials, these peaks, which we attribute to photoelectrons, were 20%–30% higher than the 10 V_ESA_ background count rate. Note that all UV and photoelectron testing includes the uncertain variable of the MCP’s detection efficiency for UV, which is likely in the range of 1%–10% but varies based on MCP manufacturing techniques.

### H. Vibration testing

**FIG. 11.**
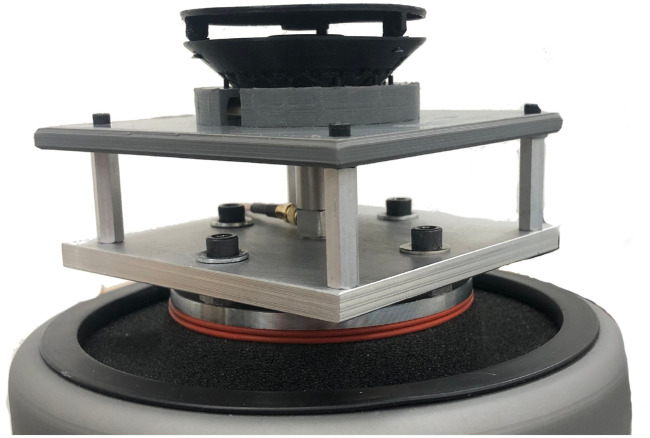
Printed ESA on vibration table.

The printed CNT-PEEK ESA was subjected to random vibration testing in two orientations, as is typical in qualification testing for space flight. We scaled the NASA GEVS (General Environmental Verification Standard) 14.1 G 20-2000 Hz random vibration qualification test profile up to 20 G rms to make a more challenging test. Figure 11 shows a photo of the ESA in “flat” orientation on the vibration table at UAF’s College of Engineering and Mines. The ESA was also tested in the shearing orientation, such that vibratory acceleration was applied in the direction transverse to the ESA symmetry axis, applying shear forces to the relatively narrow posts that support the top cap.

**FIG. 12.**
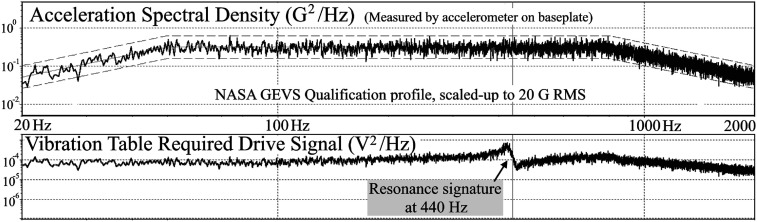
Resonance seen at ∼440 Hz on the vibration table input drive signal from the 20 g-intensified GEVS vibration profile.

Figure 12 shows the vibration frequency spectrum that was applied to the ESA. There was no damage to the ESA after 20 minutes in both orientations at 20 G rms. The ESA did show a distinct resonance in the “flat” orientation at about 440 Hz. We assume the top cap is the most likely source of this resonance by moving like a drum head, since it is only supported at three points along its outer edge.

Finally, we added 30 g (nearly 3× the CNT-PEEK ESA’s own mass) of aluminum screwed on to the top cap to simulate electronics being mounted in that location. The ESA survived a 20-minute vibration with that additional mass in the shearing orientation at 14.1 G rms.

### I. Outgassing and residual gas analysis

We were initially concerned that voids between the printed extrusion lines of plastic may cause increased outgassing or “virtual leaks” in vacuum. In practice, this has not been a problem. We have not noticed any troublesome increase in vacuum pumping time required to reach a certain pressure. The increased surface area on printed parts probably does retain some additional adsorbed water and air, but it has not been an obstacle to reaching the pressures we expect to achieve. Baking at about 100 °C has allowed these printed parts to reach the limits of UAF’s vacuum chambers, roughly 5 × 10^−8^ Torr, and we did not encounter trouble reaching ion gauge pressure readings around 1 × 10^−7^ Torr on GSFC’s cryo-pumped chamber either.

**FIG. 13.**
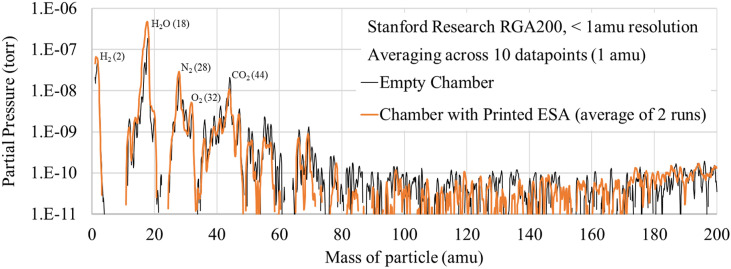
Comparing RGA scans of empty chamber and printed ESA.

The results from residual gas analyzer testing at GSFC in Fig. 13 show minimal difference between the empty chamber test case (black) and the test with the CNT-PEEK ESA inside (orange). This indicates that outgassing from the CNT-PEEK is probably not a concern. Most importantly, the measurements at the highest molecule masses are very near the RGA’s noise floor, indicating that there are very few high-mass hydrocarbons, which are the largest threat for condensing and degrading MCPs or other sensitive surfaces. The main pressure peaks all correspond to easily identifiable low-mass gases, which probably will appear in any normal RGA scan. A full outgassing analysis of condensable material should be performed in the future.

## V. CONCLUSIONS AND FUTURE DIRECTIONS

The use of CNT-filled PEEK in an AM fabrication of a realistic ESA design for scientific space flight is demonstrated to be feasible, although some concerns remain. At least for the keV electron energies we tested, the as-built CNT-PEEK ESA clearly has sufficient electrical conductivity to execute exponential voltage sweeps as needed for high-resolution science measurements. Very fast stepped voltage sweeping may still be limited, but we expect additional improvements in conductivity to make the plastic’s ESA’s R*C time constant even faster. Electron beam testing confirms that the CNT-PEEK printed ESA displays nominal energy and angle bandpass performance, even at extremely high incident electron fluxes and extreme temperatures. Electron beam tests demonstrate that the CNT-PEEK ESA displays rejection of out-of-band electrons that is far superior to that of uncoated machined aluminum. Furthermore, vacuum testing at GSFC under exposure to a beam of ∼120 nm UV photons simulating solar Ly-α intensity at Earth’s distance from the sun demonstrates that the CNT-PEEK ESA is also far superior to the uncoated machined aluminum in rejection of both directly reflected Ly-α photons and the photo-electrons that they generate within the ESA. We have tested the CNT-PEEK ESA’s electron transmission properties over wide ranges of electron beam flux and ambient temperature, finding small variations in performance. We have shown that the CNT-PEEK ESA withstands vibration loads exceeding those expected to be encountered during a space launch, namely the GEVS standard. Finally, this work demonstrates that properly cleaned CNT-PEEK printed parts do not emit easily detectable amounts of heavy molecules under high vacuum, evidence that they may be suitable for use in space flight near contamination-sensitive surfaces.

There are several areas of remaining concern, yet to be fully resolved. These include possible electrical surface charging, degradation of the PEEK under long-term space exposure, and the on-orbit thermal performance of the CNT-PEEK.

There exists a previous space-borne ESA experiment that requires consideration. That is the Solar Wind Electron Analyzer (SWEA; Sauvaud *et al.*, 2008) that was flown as part of the In situ Measurements of Particles and CME Transients (IMPACT; Luhmann *et al.*, (2008) experiment on the two Solar Terrestrial Relations Observatory (STEREO; Kaiser *et al.*, (2008) spacecraft. The SWEA instrument featured a tophat ESA, whose top cap electrode was coated with a carbon-filled teflon material called Nuflon™. Fedorov *et al.* (2011) described that, once on orbit, SWEA experienced anomalous transmission characteristics, such that solar wind electrons below about 100 eV were not transmitted to the detector as expected. After extensive analysis, the authors concluded that the Nuflon surface had developed “isolated spatial domains” that lack sufficient conductivity to carry away incident electrons. They concluded that, taken together, these domains charged the top cap to an average of ∼4 V negative and that this potential was prohibiting normal transmission of solar wind electrons, as observed. It is notable that the authors further concluded that this phenomenon was induced by the fracturing of chains of carbon grains embedded in the Teflon surface in the extremely cold conditions under which SWEA operates, always in the shadow of the STEREO spacecraft, absent the photon flux that could have neutralized acquired negative charge by photo-emission. In the context of the present paper, this result introduces a future necessity for low energy charged particle testing under cold conditions. We have only just begun addressing this charging concern with testing. At GSFC, we informally did a single energy-angle scan with a 50 eV beam. That test inspired confidence because it produced a nominal bandpass with only slight distortion compared to the 3 keV bandpasses. However, follow-up tests at UAF have yielded inconsistent results, potentially due to inadequate equipment or potentially indicating a real charging problem. The much higher beam flux at UAF vs GSFC may be the source of this difference. If charging truly is a problem, it may be necessary to reconsider the conductive plastic formula.

Thermal analysis indicates that the dark colored CNT-PEEK will become hot under solar illumination in the space environment, likely reaching surface temperatures around 100 °C. Because of PEEK’s low conductivity, it will not reliably reach thermal equilibrium with the rest of the spacecraft. This is not expected to be a problem because PEEK can survive and maintain dimensional stability in temperatures well beyond 100 °C. However, thermal expansion and warping are residual concerns, as is the question of heat transfer from the hot ESA to sensitive electrical components near it, such as the MCP detector and the high voltage power supply. It may be preferable to thermally insulate electronics to protect them from ESA heating.

AM-fabricated engineering plastics can provide several important advantages for space-borne ESA experiments. One of these, mass savings, is realizable in two ways. First, the mass density of the CNT-PEEK (1.2 g/cm^3^) is less than half that of aluminum (2.7 g/cm^3^). Second, the AM fabrication technique allows placement of material only where it is needed for functional and structural performance so that unnecessary material can be more readily omitted than in the case of conventional machining. In addition, AM fabrication has the potential to enable electron-optical geometric configurations, some as-yet unimagined, that would be difficult or impossible to implement using conventional machining techniques. Finally, additive manufacturing of conductive plastics has the potential to greatly simplify the process of flight development for ESAs or other plasma instruments. This is particularly important in consideration of deploying tens or even hundreds of instruments in a space-flight constellation scenario. The superior rejection of out-of-band electrons, photoelectrons, and directly reflected solar photons by the CNT-PEEK as compared to bare aluminum holds the potential to eliminate the need for the application of black absorptive surface coatings on the ESA electrode surfaces. Those coatings are notorious for causing significant complexity in the processing and handling of ESAs during the flight development phase. The use of printers with multiple nozzles that feed different materials admits the prospect of printing monolithic ESAs that are almost immediately ready for flight, without alignment and assembly struggles. Such simplifications in the flight hardware development process are essential to enabling constellation-type missions such as Magnetospheric Constellation (MagCon; 36 spacecraft) and Magnetospheric Constellation and Tomography (MagCat; 20 spacecraft) envisioned in the most recent US National Academy of Sciences Heliophysics Decadal Survey (2013).

A separate but related project at UAF is currently testing whether MCPs are degraded by operating in close proximity to printed PEEK. The results will inform future designs involving printed plastic MCP detector assemblies.

Degradation from exposure to the space environment is a concern for the future. We do not expect a problem on sounding rockets or low-orbiting CubeSats, but perhaps on longer missions. Penetrating ionizing radiation, atomic oxygen, or solar UV might degrade the PEEK. The main concerns would be loss of surface conductivity or loss of UV absorptivity.

Future development focuses on (1) retiring the concerns described earlier with additional testing, (2) further improving the UV rejection of the CNT-PEEK ESA, and (3) designing, manufacturing, and testing a fully monolithic CNT-PEEK ESA. We hope to develop a complete instrument based on our CNT-PEEK ESA and seek a test flight on a sub-orbital rocket or an orbiting CubeSat.

## Data Availability

The data that support the findings of this study are available from the corresponding author upon reasonable request.
